# Social inequalities in utilization of a feminist telehealth abortion service in Brazil: A multilevel analysis

**DOI:** 10.3389/frph.2022.1040640

**Published:** 2022-12-06

**Authors:** Sara Larrea, Laia Palència, Mariana Prandini Assis, Carme Borrell

**Affiliations:** ^1^ Departament de Ciències Experimentals i de la Salut, Universitat Pompeu Fabra, Barcelona, Spain; ^2^ Agència de Salut Pública de Barcelona, Barcelona, Spain; ^3^ CIBER Epidemiología y Salud Pública, Madrid, Spain; ^4^ Institut d’Investigació Biomèdica (IIB Sant Pau), Barcelona, Spain; ^5^ Faculty of Social Sciences, Universidade Federal de Goiás, Goiânia, Brazil

**Keywords:** self-managed abortion, misoprostol, mifepristone, social inequalities, abortion access, multilevel analysis, telehealth, Brazil

## Abstract

The disruption caused by the COVID-19 pandemic on health services around the world boosted interest over telehealth models of care. In Brazil, where abortion is heavily restricted, abortion seekers have long relied on international telehealth services to access abortion pills. We conducted a cross-sectional multilevel study to assess the effect of individual and contextual social factors on utilization of one such service. For the individual-level, we analyzed data from the records of abortion seekers contacting this feminist international telehealth organization during 2019 (*n* = 25,920). Individual-level variables were age, race, education level and pregnancy length. Contextual-level units were states, for which we used data from the national Demographic Census and Household Surveys. Contextual-level variables were household income per capita, adjusted net school attendance rate, percentage of racialized women and income Gini Index. We fitted five multilevel Poisson Mixed-effects models with robust variance to estimate prevalence ratios (PR) of service utilization, which was defined as receiving abortion pills through the service. We found that only 8.2% of requesters got abortion pills through the service. Utilization was higher among women who were older, white, more educated and 5–8-weeks pregnant. Independently of this, service utilization was higher in states with higher income and education access, with lower proportions of racialized women, and located in the South, Southeast and Central-West regions. We concluded that while feminist telehealth abortion initiatives provide a life-saving service for some abortion seekers, they are not fully equipped to overcome entrenched social inequalities in their utilization, both at individual and contextual levels.

## Introduction

Along with other countries in Latin America and the Caribbean, Brazil has a long legacy of colonialism and persistent socioeconomic inequalities that have diminished during the last couple of decades (1–3). This improvement has been attributed to the establishment of cross-sector welfare policies, labor formalization, increasing education access and other distributive policies (1, 3). The welfare policies established after the country's redemocratization were strengthened during the first decade of the 2000s and include the creation of the SUS (Sistema Único de Saúde), a unified healthcare system based on the principles of universality, equity, integrality and social participation (4). As a result, most of the population now has access to institutional delivery and modern contraceptive methods, some of the key reproductive health indicators (4).

However, improvements in socioeconomic and reproductive health indicators are not distributed equally in the Brazilian territory. The North and Northeast regions have a higher proportion of racialized women, particularly black and brown, and their population has generally lower educational levels and lower income (5). Higher development rates remain concentrated in the South, Southeast and Central-West regions, which is apparent in lower formal education, higher rates of unemployment and lower per capita income in the North and Northeast regions (6). Contraceptive use is higher among women in the South, Southeast and Central-West regions (7) and abortion incidence is higher among women living in the North and Northeast regions (8). Regional inequality is a problem long acknowledged by political economists in Brazil (9, 10). Its origin is attributed to the historical social formation of the regions that up to this day determines the relative population composition of each region, which has been reproduced throughout the centuries (10).

Moreover, abortion is highly restricted in Brazil, where it is only legal in cases of rape, when the pregnancy poses a risk to the pregnant person's life and when the fetus is anencephalic (11, 12). Abortion seekers often fall out of the very narrow legal grounds for abortion or, when under their scope, find various barriers to access legal services (13, 14). Many of those who cannot access legal services resort to the use of misoprostol, which is an analog of prostaglandin first introduced in the Brazilian market in 1986 to treat gastric ulcers (15). Soon after, Brazilian women discovered its abortifacient effect and started using it to self-induce abortions (15). Following this discovery, Brazilian researchers documented the use of misoprostol to induce abortions and health institutions and researchers around the world followed suit (15). Currently, misoprostol is recognized as a safe method to induce abortions with limited health professional's involvement and its use –alone or in combination with mifepristone—is the method of choice in a wide variety of contexts globally, including settings where abortion services are offered legally (16, 17).

Despite severe legal restrictions, abortion is a common practice in Brazil. It is estimated that 1 in 5 women will have at least one abortion by the time they turn 40 (8). While women with diverse socioeconomic positions have abortions, barriers to safe abortion disproportionately affect historically marginalized and impoverished population groups (18). For example, while abortion incidence is higher among women who are racialized and have lower educational levels, those same women are more likely to have unsafe abortions, to need hospitalization after a self-managed abortion, to face barriers to access post-abortion care and to be criminalized (8, 14, 18, 19). On the other hand, white, educated, and richer women resort to local private clinics that offer safe abortion services clandestinely, or travel abroad to obtain legal abortions (19, 20).

Currently, nearly 50% of clandestine abortions in Brazil are done using pills, mainly misoprostol (8). While the transition to medication abortion as the most common abortion method contributed to a decrease of abortion-related morbidity and mortality (18), misoprostol still features in the national list of substances and drugs subjected to special control (21). People cannot access abortion pills in pharmacies, thus often procuring them through vendors in the informal market and international online organizations, while risking being criminalized (19, 22, 23). International feminist organizations facilitating access to medication abortion *via* telehealth have operated in Brazil since 2004. Based online, these initiatives operate from territories where abortion is legal and deliver mifepristone and misoprostol often by mail. They also use email to provide practical and emotional support before, during and after the abortion process. Those who are in the economic position to donate are requested a voluntary contribution to access the service, the equivalent of 70–90 Euros (22, 24). The model varies slightly from organization to organization, but they have in common the use of the Internet to provide access to safe abortion information and online accompaniment while ensuring the delivery of the pills through different methods.

Social and racial inequalities in abortion access and safety have been studied in Brazil at the individual-level (8, 14, 18). Multilevel analyses have been used to examine adolescent fertility and reproductive behaviors. These analyses indicate that state-level income inequalities, coverage of reproductive health services, maternal mortality rates and regions are associated with adolescent fertility (25–27). To the best of our knowledge, there are no studies assessing the combined effect of individual and contextual variables on clandestine abortion access. Additionally, little is known about the relationship between structural inequalities and utilization of initiatives that use the Internet to increase access to safe abortion. During the last few years, and particularly with the advent of COVID-19, Internet-based abortion provision has been continuously growing both in Brazil and around the world (28, 29). With this study, we aim to contribute to the understanding of inequalities in abortion access in Brazil by assessing how individual and contextual social factors impact utilization of an online feminist service supporting self-managed abortion.

## Methods

### Study design, population and information sources

We conducted a cross-sectional multilevel study with women being individual-level units and Brazilian states being contextual-level units. For the individual-level, we used data from the records of a feminist telehealth service. Study participants were living in Brazil, requested the service between January 1st and December 31st, 2019, were less than 9 weeks pregnant and between 14 and 49 years old. Upon service request, requesters fill out an online survey containing questions to identify eligibility for medication abortion according to the latest WHO guidelines available (16). They are also asked for personal contact information, shipping address and socio-demographic data (age, educational level and race) and a donation of the equivalent of 75 Euro, which can be done *via* credit card or international bank transfer. People who cannot donate but has answered an automatic email about the donation are still offered the pills. During the study period, around 6% of those who accessed the pills through the service did not donate. A package containing one pill of 200 mg of mifepristone and 8 pills of 200 mcg of misoprostol is shipped to people who fulfill the service requirements. Instructions on how to use the pills as well as detailed information about the abortion process are provided *via* email in the requester's language. Users are instructed to take the mifepristone pill orally and to use a dose of 800 mcg of misoprostol buccally or sublingually 24 h after having taken the mifepristone. During the study period, 25,920 people in Brazil requested a medical abortion from the service and 2,121 received the abortion pills.

For the contextual level, we used state-level data from the 2010 Demographic Census and the Annual Continuous National Survey of Households of 2015, 2019 and 2020, conducted by the Brazilian Institute of Geography and Statistics (IBGE).

### Variables

#### Outcome variable

• Service utilization: Defined as having received a package with abortion pills as recorded by the service staff among all those who requested the service (yes/no).

#### Independent variables

##### Individual-level

• Age: We calculated age from the date of birth and categorized it for descriptive purposes (14–19, 20–24, 25–29, 30–34, 35–39 and 40–49 years old). For the multilevel analysis we used the original continuous variable without the categories.
• Race: Requesters were asked “among the following alternatives, do you recognize or identify yourself as color or race: white, black, brown, yellow, indigenous?” We maintained the original responses as categories for descriptive statistics and categorized responses as white and racialized for the multilevel analysis. Racialized category included brown, black, yellow and indigenous women.
• Higher educational level accessed: Requesters are asked “what is the highest level of education you have attained?” and given the following options: Incomplete/complete primary (elementary) education, Incomplete/complete secondary education (high school), incomplete/complete tertiary (technical) education, incomplete/complete university education, incomplete/complete post-graduate education, incomplete/complete doctorate. We categorized responses as basic education (up to complete elementary education), secondary education (up to complete secondary education) and university or more (incomplete tertiary education or more).
• Pregnancy length: Requesters are asked their pregnancy length in weeks and can choose a number of weeks between 4 and ≥9 weeks. Being less than 9 weeks pregnant is a requirement to access the service. We categorized pregnancy length as follows: 4 weeks, 5–6 weeks, 7–8 weeks.

##### Contextual level

• Rate of requests: We calculated the rate of requests for each state to assess if the geographic distribution of requests was homogeneous. To do so, we divided the number of requests in each state by the number of women at reproductive age in the same state and then multiplied it by 100,000. We used data of the 2010 Demographic Census.
• Percentage of service utilization: Using data from the service records, we calculated the proportion of requesters from a given state that accessed the pills through the service among requesters from the same state that requested the service.
• School attendance percentage: It is the percentage of women 18–24 years old who attend school at the appropriate level for their age group, among the total number of women in the same age group. This data was extracted from the Annual Continuous National Survey of Households-2nd quarter 2019.
• Percentage of racialized women: We calculated the percentage of women who self-identify as indigenous, black, brown or yellow, among the total population of women in each state using data from the 2010 Demographic Census.
• Gini: The Gini index measures the extent to which the distribution of income deviates from an equal distribution, where 0 represents perfect equality and 1 indicates perfect inequality. We used the Gini index of monthly income distribution of persons aged 15 years and over for the year 2015. Data was collected within the National Survey of Households 2015.
• Region: We used the IBGE classification of regions (North, Northeast, Southeast, South, Central-West).
• Household income per capita: It is the ratio between the total earnings (in Brazilian Reais, R$) and the total number of residents of a household. The source of the data was the Annual Continuous National Survey of Households – 2020. For the multilevel analysis, we divided the variable by 1,000 so that coefficients were associated to a 1,000 R$ increase in household income per capita. In November 2021, 1,000 R$ are equivalent to 161€.

### Analysis

#### Individual-level analysis

We described the distribution of our sample according to the categories of our dependent and independent variables. We also compared people who requested the service but did not use it (i.e., filled out the online survey but did not receive the pills) with those who used the service (i.e., received a package with pills), according to their socio-demographic characteristics using a Pearson's X2 test.

#### Context-level analysis

We first described all the contextual variables for states and regions. We then mapped quintiles of all our contextual continuous variables and calculated the correlations and significance levels among all the continuous variables at the contextual level. We also performed ANOVA tests (or Kruskal–Wallis tests when the hypothesis of equal variances was not fulfilled) to examine the correlation between our categorical variable regions and the contextual continuous variables.

#### Multilevel analysis

We fitted five multilevel Poisson Mixed-effects models with robust variance, with individual data in level 1 and state data in level 2, to estimate prevalence ratios (PR) of service utilization with 95% confidence intervals (CI) and variance of random effects. Model 0 included all the individual-level variables. In Models 1–5 we included all the individual-level variables and one contextual variable in each model: region in Model 1, household income per capita in Model 2, school attendance percentage in Model 3, percentage of racialized women in Model 4 and income Gini index in Model 5. In each of these models we calculated the percentage of variance explained by the inclusion of the contextual variable in the model by comparing the variance with that of Model 0.

We used STATA 14 to run our analyses and R to plot the maps.

### Ethical issues

Upon acceptance of the terms and conditions of the service, requesters consent to the use of their anonymized data for research purposes. We followed the principles of the Declaration of Helsinki on Human Research and the study was approved by the Drug Research Ethical Committee CEIm -Parc de Salut MAR, Barcelona (Code: 2018/8145/I).

## Results

### Individual-level results

Our study population included 25,920 requesters. Table 1 presents descriptive data on the individual-level variables as well as the results of our bivariate analysis on service utilization. Most requesters were between 20 and 24 years old (34.9%), self-identified as white (48.3%), had access to secondary or university education (46.5%) and were 6 weeks pregnant or less (75.3%).

**Table 1:** Socio-demographic characteristics of study participants, service utilization and *p* value of the association between socio-demographic characteristics and service utilization.

	Total		Utilization				
	Requested the service		Yes (received the pills)		No (did not receive the pills)		*p*
	N	(%)	N	(%)	N	(%)	
Variable	25,920	(100.00)	2,121	(8.18)	23,799	(91.82)	
Age (years)							0.001
14–19	6,436	(24.83)	285	(4.43)	6,151	(95.57)	
20–24	9,051	(34.92)	724	(8.00)	8,327	(92.00)	
25–29	5,075	(19.58)	524	(10.33)	4,551	(89.67)	
30–34	2,999	(11.57)	330	(11.00)	2,669	(89.00)	
35–39	1,735	(6.69)	184	(10.61)	1,551	(89.39)	
40–49	624	(2.4)	74	(11.86)	550	(88.14)	
Race							0.001
White	12,510	(48.26)	1,232	(9.85)	11,278	(90.15)	
Brown	9,262	(35.73)	621	(6.70)	8,641	(93.30)	
Black	3,276	(12.64)	216	(6.59)	3,060	(93.41)	
Yellow	673	(2.6)	45	(6.69)	628	(93.31)	
Indigenous	199	(0.77)	7	(3.52)	192	(96.48)	
Higher educational level accessed							0.001
Basic education	2453	(9.46)	76	(3.10)	2,377	(96.90)	
Secondary education	11,427	(44.09)	567	(4.96)	10,860	(95.04)	
University or more	12,040	(46.45)	1,478	(12.28)	10,562	(87.72)	
Pregnancy length							0.001
4 weeks	9135	(35.24)	466	(5.10)	8,669	(94.90)	
5–6 weeks	10,381	(40.05)	1,067	(10.28)	9,314	(89.72)	
7–8 weeks	6404	(24.71)	588	(9.18)	5816	(90.82)	

2,121 requesters (8.2%) accessed abortion pills through the service during the study period. There was a clear utilization gradient based on age: 11.9% of people in the 40–49 category received the pills through the service, but only 4.4% of people 14–19 years old did. The proportion of white women who used the service almost triples that of indigenous women who did and is 3 percentage points higher that the proportion of brown, black and yellow women who used the service. Women with university education received the pills 4 times more than their peers with basic education. People who were between 5- and 8-weeks pregnant received the pills at double the rate than those who were earlier in pregnancy. The association between service utilization and all individual-level variables was statistically significant.

### Context-level results

The distribution of context level variables by state and region is presented in Table 2. Brazil has 27 states that are organized in 5 regions. The states' population size varies widely: the state with the smallest population is Roraima, which has 631,181 inhabitants and 181,783 women at reproductive age, while São Paulo, the largest state in the country, has more than 46 million inhabitants and more than 12 million women at reproductive age.

**Table 2:** Context-level variables by region and state.

Region and State	Population^1^	Women at reproductive age (14–49 years)^2^	School attendance percentage^3^	Percentage of racialized women^4^	Household income *per capita* (R$)^5^	Gini index^6^	Number of requests per 100,000 women at reproductive age	Utilization (% of requesters that received the pills through the service)
Brazil	211,755,692	58,720,495	29.7	51.5	1,380	0.491	44.3	8.17
North Region	18,672,591	5,411,780	23.2	76.0	-	0.473	20.6	3.49
Rondônia	1,796,460	519,713	30.6	64.3	1,169	0.452	25.4	3.79
Acre	894,470	258,680	27.8	75.9	917	0.5	15.5	0
Amazonas	4,207,714	1,206,738	24.3	77.9	852	0.476	19.5	3.4
Roraima	631,181	181,783	26.8	78.0	983	0.5	41.8	7.89
Pará	8,690,745	2,532,687	18.4	77.6	883	0.465	17.2	2.98
Amapá	861,773	257,008	34.5	75.0	893	0.457	27.2	0
Tocantins	1,590,248	455,171	27.2	75.1	1,060	0.504	28.1	5.47
Northeast	57,374,243	16,531,575	22.7	70.0	-	0.484	21.6	5.57
Maranhão	7,114,598	2,067,949	20.2	77.4	676	0.506	13.0	2.6
Piauí	3,281,480	943,623	28.1	75.2	859	0.505	20.7	2.56
Ceará	9,187,103	2,647,793	25.8	67.3	1,028	0.453	22.8	6.12
Rio Grande do Norte	3,534,165	1,002,710	26.2	58.0	1,077	0.503	18.2	7.96
Paraíba	4,039,277	1,147,349	25.1	59.3	892	0.51	22.2	7.06
Pernambuco	9,616,621	2,752,837	23.1	62.5	897	0.492	26.0	6.98
Alagoas	3,351,543	988,690	17.1	68.2	796	0.438	21.6	7.48
Sergipe	2,318,822	685,381	22	71.6	1,028	0.46	20.7	5.63
Bahia	14,930,634	4,295,243	20.6	77.6	965	0.481	23.2	4.41
Southeast	89,012,240	24,099,986	32.5	44.0	-	0.477	59.6	9.13
Minas Gerais	21,292,666	5,777,126	29.7	54.1	1,314	0.478	50.7	8.81
Espírito Santo	4,064,052	1,110,013	29.2	57.1	1,347	0.471	43.5	7.45
Rio de Janeiro	17,366,189	4,661,660	33	51.6	1,723	0.454	59.0	10
São Paulo	46,289,333	12,551,187	34	35.2	1,814	0.47	65.3	9.05
South	30,192,315	7,992,461	36.8	20.8	-	0.45	53.1	8.93
Paraná	11,516,840	3,099,169	37.1	28.9	1,508	0.459	51.6	8.76
Santa Catarina	7,252,502	1,951,305	40.2	15.4	1,632	0.419	59.4	8.19
Rio Grande do Sul	11,422,973	2,941,987	34.2	16.2	1,759	0.487	50.4	9.7
Central-West	16,504,303	4,684,693	37.3	57.5	-	0.498	47.9	7.76
Mato Grosso do Sul	2,809,394	764,065	31.4	52.0	1,488	0.479	45.3	8.38
Mato Grosso	3,526,220	977,797	32.4	62.2	1,401	0.445	43.2	6.4
Goiás	7,113,540	2,010,623	38	57.6	1,258	0.436	39.8	5.25
Distrito Federal	3,055,149	932,208	45.4	57.1	2,475	0.555	72.3	11.28

Sources: (1) IBGE, Population estimations 2020. (2) IBGE, Population projections 2018. (3) IBGE, National Survey of Households- 2nd quarter 2019. (4) IBGE, Demographic Census, 2010. (5) National Survey of Households-2020. (6) National Survey of Households-2015.

Other regional characteristics also differ widely. For example, the North and Northeast regions have the highest proportions of racialized women (76% and 70% respectively), the lowest school attendance rates (23.2% and 23.7% respectively) and the states with the lowest income per capita (676R$ for Maranhão, 796 R$ for Alagoas and 852R$ for Amazonas). The Central-West region has the highest school attendance rate (37.7%), and the highest income inequality (Gini index: 0.498). The South region has the lowest proportion of racialized women (20.8%) and the lowest income inequality in the country (Gini index: 0.450).

**Figure 1:**
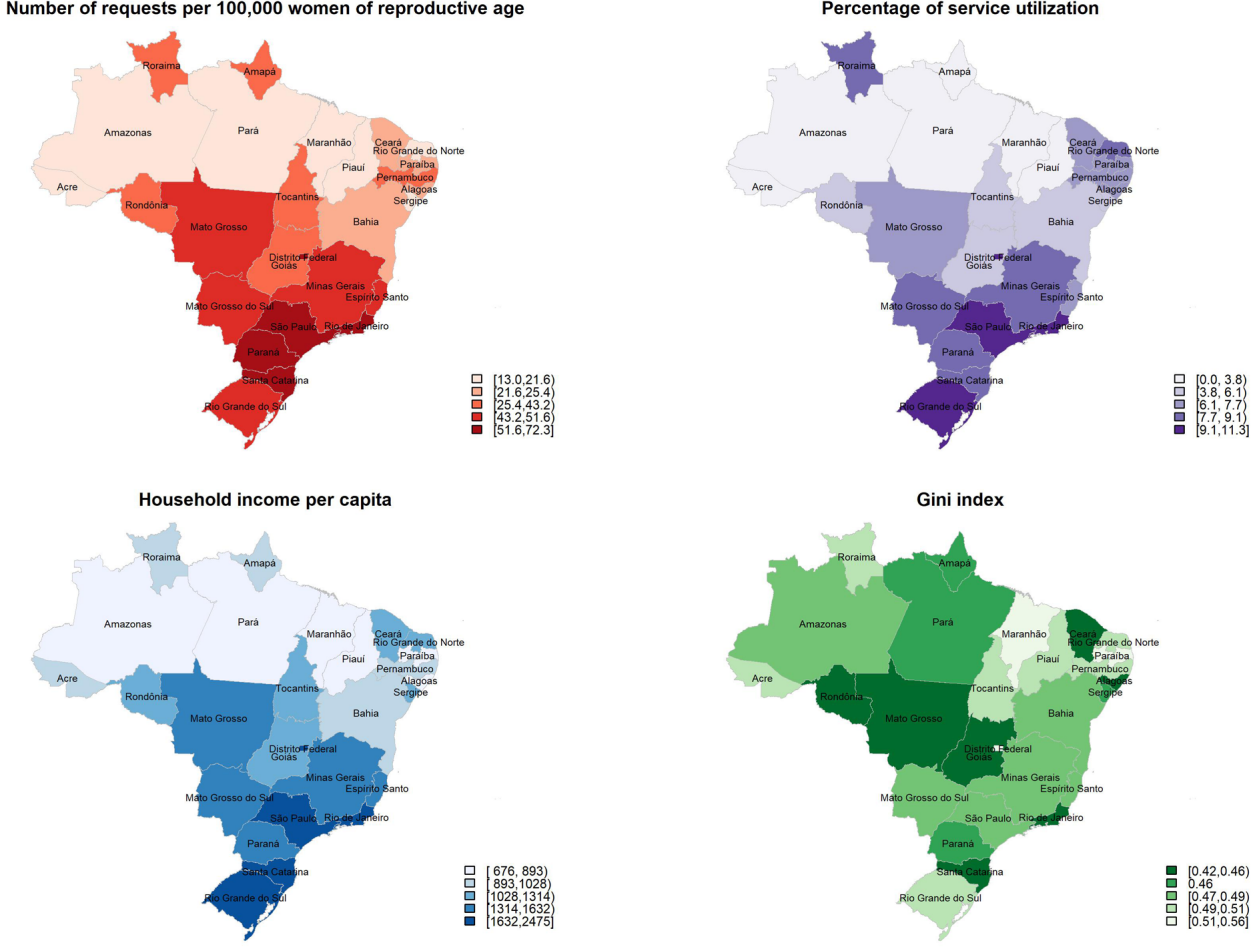
Number of requests per 100,000 women at reproductive age, percentage of service utilization, household income per capita and Gini index according to Brazilian states.

A selection of variables in Table 2 are mapped in Figure 1, which shows the geographic distribution of requests, percentage of service utilization, household income per capita and income Gini index by state. The first three maps show the same pattern: the number of requests, the percentage of service utilization and the household income per capita are higher in the states of the South, Southeast and Central-West regions and lower in the North and Northeast regions. The maps of education level and percentage of racialized women (not shown) follow the same pattern. The map of the Gini index highlights the slight differences in income inequality by regions and does not follow the same pattern than the other maps. Generally, inequality is the highest in Central-Western and Northeastern states and the lowest in the Southern states.

The relationship between contextual variables is shown in Table 3. There is a positive and statistically significant correlation between utilization and household income per capita (*r* = 0.727) and adjusted net school attendance rate (*r* = 0.409). This means that the probability of using the service is higher in states with higher income per capita and with higher school attendance percentages. We also found an inverse correlation (*r* = −0.659) between utilization and the percentage of racialized women, meaning that the probability of accessing the pills through the service is lower in states with higher proportions of racialized women. There is also a statistically significant relationship between utilization and region, meaning that there is a higher probability of service utilization in the South, Southeast and Central-West regions. Utilization is not statistically significantly correlated with the Gini index.

**Table 3:** Correlations and their *p*-values between variables of the contextual level.

	Utilization	Adjusted net school attendance rate	Percentage of racialized women	Household income per capita	Gini Index
Utilization	1	** **	** **	** **	** **
Adjusted net school attendance rate	0.4095	1	** **	** **	** **
	*0*.*0339*	** **	** **	** **	** **
Percentage of racialized women	−0.659	−0.6283	1	** **	** **
	*0*.*0002*	*0*.*0004*	** **	** **	** **
Household income per capita	0.7273	0.8177	−0.6877	1	** **
	*<0,0001*	*<0,0001*	*0*.*0001*	** **	** **
Gini Index	0.0586	−0.0298	0.2702	0.0784	1
	*0*.*7716*	*0*.*8828*	*0*.*1728*	*0*.*6974*	** **
Region^a^	*0*.*0018*	*0*.*0001*	<0.0001	*0*.*0007*	*0.5949*

^a^ Region is a categorical value so the *p*-value is the one for the ANOVA test with the other variables.

We also found positive correlations between the school attendance percentage and income and region, meaning that school attendance rate is higher in states with higher income and in some regions. There was a negative association between the proportion of racialized women and school attendance rate and income per capita, meaning that in states where the proportion of racialized women is higher, school attendance rate and income per capita are lower.

### Multilevel results

Table 4 shows the results for our multilevel models. The null model indicates a positive association between utilization and the individual-level variables of race, higher educational level accessed and age. This means that the probability of service utilization is 21% higher for white women than for racialized women; 2.7 times higher for women who accessed university education and 69% higher for those who attended secondary education than for women with basic education; and increases 1% for the increase of each year of age. For pregnancy length the association is positive, meaning that when compared with those who are 4 weeks pregnant, people between the 5th and the 8th week of pregnancy use the service 86%–87% more.

**Table 4:** Prevalence ratios (PR) of service utilization with 95% confidence intervals and residual variance for multilevel logistic regressions.

	Model 0	Model 1	Model 2	Model 3	Model 4	Model 5
	PR (IC 95%)	PR (IC 95%)	PR (IC 95%)	PR (IC 95%)	PR (IC 95%)	PR (IC 95%)
Race						
Racialized	1	1	1	1	1	1
White	1.21 (1.16–1.27)	1.20 (1.14–1.25)	1.21 (1.16–1.27)	1.20 (1.15–1.26)	1.20 (1.14–1.26)	1.21 (1.15–1.27)
Higher educational level accessed						
Basic education	1	1	1	1	1	1
Secondary education	1.69 (1.34–2.12)	1.69 (1.34–2.12)	1.69 (1.34–2.12)	1.69 (1.34–2.12)	1.69 (1.34–2.13)	1.69 (1.34–2.12)
University or more	3.73 (3.05–4.57)	3.75 (3.06–4.70)	3.72 (3.04–4.54)	3.73 (3.05–4.56)	3.74 (3.05–4.58)	3.73 (3.05–4.57)
Age	1.01 (1.01–1.02)	1.01 (1.01–1.02)	1.01 (1.01–1.02)	1.01 (1.01–1.02)	1.01 (1.01–1.02)	1.01 (1.01–1.02)
Pregnancy length						
4 weeks	1	1	1	1	1	1
5–6 weeks	1.87 (1.70–2.05)	1.87 (1.70–2.06)	1.87 (1.70–2.05)	1.87 (1.70–2.05)	1.87 (1.70–2.05)	1.87 (1.70–2.05)
7–8 weeks	1.86 (1.65–2.09)	1.86 (1.65–2.09)	1.86 (1.65–2.09)	1.86 (1.65–2.09)	1.86 (1.65–2.09)	1.86 (1.65–2.09)
Region						
North		1				
Northeast		1.53 (1.06–2.22)				
Southeast		2.43 (1.76–3.35)				
South		2.21 (1.58–3.09)				
Central–West		2.00 (1.30–3.07)				
Household income per capita (1,000 Reais)			1.59 (1.27–2.00)			
Adjusted net school attendance rate				1.02 (1.00–1.04)		
% of racialized women					0.992 (0.98–0.99)	
Gini Index (2015)						0.052 (0.00–4.29)
Variance	0.232	0.015	0.011	0.098	0.072	0.237
% variance reduction with respect to model 0		93.4	95.1	57.7	68.8	−2.2

Results for Model 1 show a positive association between service utilization and region, meaning that when compared with the North region, the probability of using the service is 53% higher in the Northeast, 100% higher in the Central-West, 121% higher in the South and 143% higher in the Southeast region. Region accounts for 93.4% of the state-level variance in utilization. Model 2 indicates that utilization increases 59% (95% CI 27%–100%) for each increase of 1,000 R$ in household income per capita, and 95.1% of the variance in state-level utilization is attributed to income on its own.

Model 3 indicates that utilization is also positively associated with the adjusted net school attendance rate, meaning that the probability of using the service increases 2% (95% CI 0.1%–4.0%) for each percentage point increase in school attendance. Independently, school attendance rate accounts for 57.7% of the state-level variance in utilization. Model 4 indicates a negative association between utilization and percentage of racialized women, meaning that the probability of using the service decreased 1% for each point increase in percentage of racialized women. Model 5 did not indicate an association between utilization and income inequality.

## Discussion

We found evidence of both individual and contextual inequalities in utilization of the telehealth abortion service we analyzed. The prevalence of service utilization was higher among women who were older, white, with higher levels of education and between 5 and 8 weeks pregnant. Independently of individual characteristics, service utilization was higher in states with higher incomes, higher percentage of educated women, lower percentage of racialized women and located in the South, Southeast and Central-West regions. Income inequality was not associated with utilization.

Our analysis of requests showed that women of all age groups, races and education levels resorted to the telehealth service when seeking an abortion, which is in line with previous studies showing that Internet is an important source of information and abortion access in Brazil (24, 30). However, women from regions that are richer, have higher education levels and lower proportions of racialized people were more likely to request the service, which contrasts with the higher abortion incidence in the North and Northeast regions (8). One possible explanation for this territorial inequality are the differences in Internet access: while more than 70% of Brazilians have some kind of Internet connection, the coverage and quality of the Internet vary widely according to region and income. Most of the North and Northeast regions depend on satellite or mobile connections and, while 99% of households with higher socioeconomic status are connected to the Internet, only 43% of households of lower socioeconomic status have internet access (31). Thus, differences in Internet access could act as the intermediate mechanism by which geographic and economic inequalities determine access and utilization to this telehealth service.

We also found that only a small proportion of requesters (8.2%) actually obtained abortion pills through the service, which generally indicates poor access to the pills and strong barriers to service utilization. Moreover, among the small percentage of requesters who were able to access the pills through the service, most were white, 5–8 weeks pregnant and educated, indicating the existence of entrenched social inequalities in this service utilization. While the organization has a policy of providing the service also to those who cannot donate, only 6% of users received the pills without donating. One possible explanation for the small proportion of people with lower economic status utilizing the service is that those with difficulties to donate do not believe that they will be able to access the service without making a payment, which would indicate that there are symbolic barriers deterring people from continuing communicating with the service after filling out the service request. From our perspective, both the amount of the requested donation and the requirement of an international payment method – credit card or bank transfer- could also act as barriers for this service utilization. Supporting our hypothesis, a study in Kenya found that some payment methods can constitute barriers to access and can trigger delays in abortion services utilization (32). Regarding the amount of the requested donation, in 2021 it was equivalent to 50% of a monthly minimum salary in Brazil, which makes the donation impossible to afford for most women and would explain why many of them do not follow-up on the communication after having requested the service.

Similarly, in line with previous studies, we found that older women accessed the service at higher rates than their younger peers. Being young can be a barrier for abortion access in several ways. For example, in a review of the regional evidence on pregnancy and abortion among girls 15 years old or younger, authors found that girls and young women face particular barriers to access legal abortion services, such as the lack of clarity on clinical protocols for underage women, as well as norms that require parental consent or judicial authorization when parents do not consent or are not involved in the process (33). Regarding this specific service, younger women may not have information about its existence, lack access to private devices to connect to the Internet, face more difficulties to gather the money for the requested donation and to find a way to transfer it internationally. In any case, young women who do not get abortion support will get the abortion by other means and may be at higher risk of being criminalized, as research in the region has shown (34, 35).

Race and educational level are also important determinants of utilization in this abortion service. In Brazil, abortion incidence is higher among racialized women and women with lower socioeconomic status (8), but they face more barriers to access safe abortion methods and post-abortion care (14). They are also more often targeted by the criminal justice system (14, 18). Our results show that this pattern still stands for those who resort to this feminist service, thus revealing that structural inequality operates both in the formal healthcare system and in civil society initiatives and highlighting the need for policies that target entrenched racial inequalities in abortion access.

Service utilization was also associated with pregnancy length, with women who are between 5 and 8 weeks pregnant utilizing the service more often. The fact that women who are very early in their pregnancies use this service at lower rates could be explained by some people requesting the service before confirming the pregnancy, combined with a high proportion (between 11% and 30%) of early pregnancies ending in natural miscarriages (36) or because being early in the pregnancy translates into having more time to find local solutions. However, those who are 9 weeks pregnant or more and thus do not fulfill the service requirements, are likely to face more difficulties accessing safe abortion elsewhere, which could cause further delays or even mean they will have to carry the pregnancy to term, as has been demonstrated by other studies in the region (37, 38).

Our context-level analysis showed that the probability of using the service increases when income and school attendance are higher at the state-level and is lower in states with higher proportions of racialized women. Furthermore, some of these variables are also correlated among them, confirming the compounded nature of inequality and exclusion shown by previous research showing that under-served communities are also often racialized, with lower income and access to formal education (9, 39).

The results of our multi-level analysis showed that while service utilization is associated with individual characteristics, most of its state-level variability is explained by contextual variables such as region and state income per capita, which account for more than 90% of the variance in state-level utilization. Thus, our results indicate that social inequalities in abortion access are not a unique feature of this service, nor a result of its users' individual characteristics, but they also reflect the broader social context in which Brazilians seek abortion services. While an important promise of medication abortion provision is to dismantle geographic barriers in abortion access (40), we showed that inequalities based on age, race, income, and formal education, can be reproduced even in a remote, Internet-based service. On top of unequal access to internet and income, other reasons such as lower levels of health literacy, familiarity with online transactions and empowerment to act on their reproductive desires may explain why women from the most disenfranchised population groups and territories are not able to access abortion pills through this service.

The evidence of inequalities in the utilization of this abortion service highlights, first, the relevance and need of working to improve equity in access to abortion support. Second, our research points to the pressing need to consider the local context in structuring services so that telehealth models of care can fulfill their aim of confronting contextual and individual inequalities in abortion access, instead of reproducing them. Much can be learned from feminist initiatives that have implemented specific strategies to tackle social inequalities in access to their support. For example, some feminist organizations use phones, rather than Internet, to provide abortion information, which amplifies access to disadvantaged population groups. Others organize in-person meetings with large groups of women to ensure that all the necessary information as well as the medication can be delivered in one single opportunity (37, 41, 42). While not all feminist local initiatives in the region request a payment or donation in exchange for their support, those who do are able to establish an amount that is better suited for the specific context they work in, making the service affordable for at least most people in their own territories. Other activists have established alliances with health professionals to secure the people they support will have access to the public health system if needed and desired (43).

More research is necessary to understand how social inequalities in access to remote abortion services operate in different contexts, including less restrictive and less unequal settings. In terms of services and policies, our results suggest that remote Internet-based initiatives supporting abortion access are not sufficient to reach the most marginalized social groups in a very unequal context such as Brazil, which underscores the need for local and in-person provision of abortion services or for remote alternatives that are specifically tailored to local contexts. Thus, to secure equity in safe abortion access, abortion decriminalization and provision of public abortion care are needed.

### Strengths and limitations

To our knowledge, this is the first multilevel analysis seeking to explain social inequalities in feminist telehealth abortion services. Moreover, the large study population made it possible to arrive to sound conclusions. Thus, our study contributes with fresh data on the relationship between social contexts, individual characteristics, and inequality in safe abortion services utilization, which is the study's major strength.

However, the study's population was not representative of the Brazilian population but only of women who requested one specific service, which caused a selection bias as shown in the results. Also, interpretations regarding the analysis of the pregnancy length in our study should be cautious: Because we found a high percentage of missing cases in the data on last menstrual period, we used limited data coming from a self-assessment of pregnancy length in number of weeks, which due to the characteristics of the service, might reflect an information bias.

### Conclusion

Our study shows that while feminist telehealth abortion services, such as the one we analyzed, secure safe abortion access for some people in highly restrictive contexts, they are not equipped to overcome entrenched social inequalities in safe abortion access. Individual-level inequalities in service utilization are associated to age, education level and race. Contextual-level inequalities are associated to region, income per capita, percentage of racialized women and schooling. Thus, our findings suggest that telehealth abortion services need to structure their models of care with specific attention to how local and structural inequalities interact with abortion provision. In addition, large scope measures, such as decriminalization and local provision of public abortion care, are necessary to secure equity in safe abortion access.

## Data Availability

The datasets presented in this article are not readily available because of security concerns of the organization. Requests to access the datasets should be directed to slarrea80@gmail.com.
